# Editorial: Evolutionary Perspectives on Human Growth and Development

**DOI:** 10.3389/fendo.2021.672452

**Published:** 2021-03-17

**Authors:** Benjamin C. Campbell

**Affiliations:** University of Wisconsin–Milwaukee, Milwaukee, WI, United States

**Keywords:** evolutionary biology, growth and development, life history, fetal development, childhood

The 10 articles in this special topic represent the fruits of an evolutionary approach to human development that spans from fetus to young adults, and across subsistence and industrialized populations. In doing so it touches on the genetic basis of human growth and development and the impact of larger environmental and social conditions. The collection will appeal to those interested in an understanding of the developmental pattern of our slowly developing and long-lived species and its variations. Below I highlight and integrate crucial elements from each of the articles in the special topic and point toward future research.

In *Why and How Imprinted Genes Drive Fetal Programming*, Crespi incorporates evolutionary ideas of genomic conflict and imprinted genes into the well-established notion of fetal programming for postnatal growth and development. Crespi‘s call for the inclusion of imprinted maternal genes in longitudinal studies of the effects of low birthweight and subsequent catch-up growth echoes through the other contributions.

In *Timing of the Infancy-Childhood Transition in Rural Gambia*, Bernstein et al. use patterns of weight for age z scores to place the infant-child transition among children in the Gambia at 9 months, some 3 months earlier than similar results in the U.K. This runs counter to ideas that undernutrition leads to extended infant growth. Instead, it suggests that infant growth may be cut short to allocate energy for immune development, a life history trade-off that deserves more attention in the future.

Turing to middle childhood, in *DHEAS and Human Development: An Evolutionary Perspective*, Campbell focuses adrenarche as an endocrinological marker. He argues that DHEAS acts at the IGF-1 receptor, giving it a role brain in development starting with the 5-8 transition. Given evidence that IGF-1 is stimulated by animal protein in the diet, the roots of human middle childhood may go back to the origins of meat consumption with the Genus Homo.

In their contribution, *Emerging Adulthood, A Pre-Adult Life History Stage*, Hochberg and Konner point to continued brain development after and lasting into the mid-20s, suggesting young adulthood as biologically based rather than socially constructed. Chimpanzee brain maturation ends at puberty, pointing to a unique human life history stage that needs to be characterized more fully in terms of endocrinology and its relationship to earlier development.

Young adulthood is also marked by peak bone mass as explored by Kralick and Zemel, in *Evolutionary Perspectives on the Developing Skeleton and Implications for Lifelong Health*. In fact, Kralick and Zemel suggest that the most important determinant of osteoporosis for older women life may be low peak bone mass. Bone mass is diminished by a sedentary lifestyle. Thus, osteoporosis is not only an evolutionary disease, but changes in activity with the advent of agriculture may have been a key factor.

The importance of the agriculture revolution to modern human growth and development is central to Wells and Stock in *Life History Transitions at the Origins of Agriculture: A Model for Understanding How Niche Construction Impacts Human Growth, Demography and Health*. The article makes a forceful case for the selective impact of the agricultural revolution human growth and development through its effects on immune defenses against infectious disease. This intriguing idea deserves much future consideration in understanding growth and development in subsequent populations as well as the paleopathology of juveniles at the origins of civilization.

Little‘s contribution, *Evolutionary Strategies for Body Size* focuses on what is known about factors contribution to variation in human body size in populations outside the bounds of civilization. Variation in phenotypes such the short statured pygmies of the Congo and the long lean Turkana pastoralist of Kenya are well documented. The availability of non-invasive sample collection and assay techniques means that future research may now focus on the underlying physiological growth processes.

At the other end of the environmental spectrum, in *Sexual Dimorphism of Size Ontogeny and Life History*, German and Hochberg consider differences in height over the life span across a set of national populations. Not only does sexual dimorphism emerge with puberty, but also the authors find it is maximal under good environmental conditions. Importantly, these results hold across a set of subsistence populations as well. In other words, male growth is more responsive to environmental variation than females, and in ways that are likely derived from developmental adaptations to energy constraints.

The final two papers take the topic deeper into environments not anticipated in human evolution and more fully into the realm of global public health. *People Are Taller in Countries With Better Environmental Conditions* by German et al. demonstrates differences in adult height across OCED countries as a function of modern environments, including air pollution, income inequality and stress, with males more affected than females. It will be important to elaborate how these environmental factors map onto the those experienced during our evolution history if we are to understand future developmental outcomes and safeguard human health in the years to come.

Finally, in *Nutrition Justice: Uncovering Invisible Pathways to Malnutrition*, Hanieh et al. bring attention to social and political factors contributing to the double burden of disease in marginalized populations. But at the core of their argument is an evolutionary perspective. That underfed babies exhibit growth faltering and stunting leading to metabolic complications and disease as adults IS an evolutionary adaptation for survival. One that represents the impact of environmental fluctuation during human evolutionary history in shaping human life history. It also brings us back full circle to the genetic underpinnings of fetal programming discussed by Crespi.

Together these articles demonstrate the integrative nature of an evolutionary approach to human growth and development. Though we divide growth and development into stages, each stage is related to the next, and they are all linked to fetal development. Thus, despite its responsiveness to environmental factors, the developing human body has a remarkable capacity to return to a growth trajectory set up in utero. It is a tragedy when social conditions not only disrupt that trajectory, but also cause us to lose sight of it in the first place.

## Author Contributions

The author confirms being the sole contributor of this work and has approved it for publication.

## Conflict of Interest

The author declares that the research was conducted in the absence of any commercial or financial relationships that could be construed as a potential conflict of interest.

